# Order Batching in Warehouses by Minimizing Total Tardiness: A Hybrid Approach of Weighted Association Rule Mining and Genetic Algorithms

**DOI:** 10.1155/2013/246578

**Published:** 2013-06-20

**Authors:** Amir Hossein Azadnia, Shahrooz Taheri, Pezhman Ghadimi, Muhamad Zameri Mat Saman, Kuan Yew Wong

**Affiliations:** ^1^Department of Manufacturing and Industrial Engineering, Faculty of Mechanical Engineering, Universiti Teknologi Malaysia, Johor Bahru, 81310 UTM Skudai, Malaysia; ^2^Department of Computer Science, Faculty of Computer Science, Universiti Teknologi Malaysia, Johor Bahru, 81310 UTM Skudai, Malaysia; ^3^Enterprise Research Centre, University of Limerick, Limerick, Ireland

## Abstract

One of the cost-intensive issues in managing warehouses is the order picking problem which deals with the retrieval of items from their storage locations in order to meet customer requests. Many solution approaches have been proposed in order to minimize traveling distance in the process of order picking. However, in practice, customer orders have to be completed by certain due dates in order to avoid tardiness which is neglected in most of the related scientific papers. Consequently, we proposed a novel solution approach in order to minimize tardiness which consists of four phases. First of all, weighted association rule mining has been used to calculate associations between orders with respect to their due date. Next, a batching model based on binary integer programming has been formulated to maximize the associations between orders within each batch. Subsequently, the order picking phase will come up which used a Genetic Algorithm integrated with the Traveling Salesman Problem in order to identify the most suitable travel path. Finally, the Genetic Algorithm has been applied for sequencing the constructed batches in order to minimize tardiness. Illustrative examples and comparisons are presented to demonstrate the proficiency and solution quality of the proposed approach.

## 1. Introduction

Based on ELA/AT Kearney [1], about twenty percent of the logistic costs of the surveyed companies were incurred due to warehousing in 2003. A vital part of the logistics system of a company is involved with its warehouses. Optimization of operations within every facility must be considered as an important part of its policies in order to promptly deliver goods or services to its customers at the least cost [2]. Responsiveness is a critical success factor in a warehousing system. Product movement within a warehouse can be facilitated effectively by consolidating orders into batches which can be done before picking customer orders [3].

In order picking systems, certain due dates are assigned to customer orders which should not be violated. In order to avoid production delays, item retrieval from the warehouse needs to be done at its appropriate time. In these cases, the tardiness of customer orders should be involved with order batching instead of using the total processing time as a measure for the solution quality [4]. Henn et al. [5] defined the tardiness of a customer order as the positive value between the completion time of a customer order with its due date. The time that an order picker finishes her/his tour of gathering all required items and comes back to the starting point is called completion time. Obviously, one of the factors that can influence the completion time is the processing time of the orders. According to the above explanation, reducing the processing time and travel time for constructing a batch is an important fact in reducing the costs and minimizing delays of the customer responses. On the other hand, not considering the order's due date in the process of order batching can cause a huge dissatisfaction in customer expectations regarding the efficient responsiveness of the company. For explanation purposes, assume that we have some orders with different due dates, considering just processing time or traveling time in the process of order batching regardless of their due dates may consolidate orders with different due dates in a same batch. So, this issue may cause a problem for meeting customers' demand in a certain due date. Consequently, it is highlighted that order due dates should be considered as an important factor for order batching.

Based on the above illuminated problem, this research paper attempts to develop a novel model to overcome the aforementioned issue in the multiple parallel aisles with manual order picking system. In our proposed model, it was assumed that orders with similar items are grouped together. Therefore, batching orders with higher resemblance can reduce the order pickers' traveled distance. Also, for considering the due dates in our model, the due date of each order is defined as a weight parameter in calculating the association between orders in terms of support. The association information integrates the bathing process with various data regarding each order. For calculating the association between orders in terms of support, mining association rules with weighted items (MINWAL) were utilized. Then, a clustering approach based on a binary integer programming model has been used in order to maximize the customer demand association which is called support. Next, the Genetic Algorithm (GA) has been utilized to solve the Traveling Salesman Problem (TSP) to minimize the travelling time for collecting all items in a constructed batch. This process was followed by a batch sequencing process which utilized a GA in order to minimize the average tardiness of all orders. This paper copes with integrating batch ordering, picker routing, and the batch sequencing problem regarding orders' due date in order to minimize the average tardiness of all orders. We believe that the proposed approach, namely, ATGH is not considered in any existing published paper. In the next section, a comprehensive literature survey is presented in order to review the existing research activities related to the current research paper. 

## 2. Literature Review

### 2.1. Order Batching Problem

Order picking is an action which takes place to fulfill a customer demand (internal/external) that is normally done by retrieving items from buffer locations inside the warehouse [6–8]. In practice, there are two types of systems for order picking: systems which are totally manual and involved with human order pickers and the systems that are entirely computerized in case of retrieving items from the warehouse. Picker-to-parts and parts-to-picker systems can be categorized as the systems that belong to the first group of warehousing systems [5, 9]. Picker-to-parts systems are the most commonly used systems in warehousing in which picking the items is done by an operator that drives or walks along the aisles [10, 11]. This scenario is different in parts-to-picker systems. Order pickers do not move in this system and are located in the depot. Pallets or bins (unit load) from the warehouse are retrieved by automated storage and retrieval systems (AS/RS) and delivered to them at the depot. After that, the requested items are detached by the operators stationed in the depot and the pallets or bins are returned to its spot in the warehouse by the AS/RS [5].

In a large amount of orders, a single order picking policy might be applied in which one order can be picked in each picking tour. However, in a small amount of orders, picking loads in a single picking tour can lead to a decrease in travel times [12]. Order batching is classified as a NP-hard problem which can be done to improve warehouse efficiency by reducing operational costs [12, 13]. Thus, many heuristic algorithms are proposed in the literature which help to solve this problem. Savings and seed algorithms are the most commonly used order batching heuristics in the case of manual picking systems. Seed algorithms were introduced by [14] in which a seed order would be selected for each batch. With respect to the picking device capacity, other orders would be assigned to the seed orders. Clarke and Wright [15] first developed the savings algorithms inspired by a vehicle routing algorithm. This algorithm works based on the saving that would be achieved in travel distance or travel time. After that, a pair of batches would be selected iteratively by the algorithm and combined with each other until a capacity constraint threshold is reached that stops combining of the batches.

In the current literature review, it was tried to review all existing papers which are involved in order batching problems considering the minimization of due dates, total travel distance, and processing time. First, Vinod [16] suggested that integer programming can be used as a flexible method for grouping any kind of objects. Armstrong et al. [17] also presented an integer programming model based on predetermined batch sizes considering proximity batching. 

They tried to minimize the total processing time of all batches. Ratliff and Rosenthal [18] tried to minimize travel distance based on a procedure which helps to find the best picking tour traveled. By means of a distinguished TSP, a tour was formed in a 50 aisles warehouse. Kusiak et al. [19] used a quadratic integer programming model to obtain batches from eight orders. In their proposed model, it was tried to minimize the total sum of distances. 

One of their limitations was related to their model range of applicability which is not well suited for large numbers of orders. Gibson and Sharp [20] presented an order batching procedure integrated with a computer simulation which resulted in minimization of travel distance for each tour. Rosenwein [21] utilized Centre of Gravity (COG) and Minimum Additional Aisle (MAA) for order batching in order to measure travel distance. It was shown that the MAA metric performed better than the COG metric regarding travel tour generation. Gademann et al. [22] targeted to minimize the total travel time for all batches using a branch-and-bound algorithm. They tested their model by several test sets with a maximum order number of 32 which were compared together regarding their CPU times. Hsu et al. [3] applied Genetic Algorithms (GAs) to deal with order batching problems with any kind of configuration. In their proposed approach, they endeavored to minimize the total travel distance. In another research activity done by Chen and Wu [23]; data mining and integer programming were combined to form a solution approach regarding the order batching problem. They have tried to maximize the similarity between orders in a batch in terms of support without considering orders' due dates. Based on the presented results which was attempted to compare first-come first-served (FCFS), MAA, COG, and Gibson and Sharp's method (GSM), the authors stated that the proposed approach is effective in solving the order batching problems in terms of reducing traveling distances. Based on the analysis done by Gademann and van de Velde [24], the authors stated that the order batching problem is NP-hard to a great extent provided that no batch has more than two orders. They developed a branch-and-price algorithm to formulate the problem in order to minimize the total travel distance. Three years later, a mixed-integer programming model was developed by Bozer and Kile [25] in order to minimize the length of trips or travel distance. They stated that acquiring minimum travel distance for a large number of orders requires substantial computational time. Kulak et al. [26] proposed a solution approach to reduce the mathematical burden of order batching and picker routing problems. They proved the efficiency of their cluster-based tabu search approaches by running several test examples. They have focused on minimizing the travel distance between locations.

In practice, certain completion due dates are assigned to customer orders. For instance, in distribution warehouses, the scheduled departure of trucks has to be done by certain due dates to ensure the on time delivery of the requested items to the customers [22]. In material warehouses, avoiding production delays can be guaranteed, if retrieving the items from the warehouse is done based on their schedules. In these cases, assessing the tardiness of the customer orders needs to be focused in the process of batching customer orders into picking orders [4]. The tardiness is the difference between the order finish time and the due date, if this difference is positive. So, it is very important to consider the orders' due dates when batching processes are being done. To our knowledge, there are few papers that consider due dates or minimize tardiness in their model. Elsayed et al. [4] and Elsayed and Lee [27] focused on developing minimization models of order tardiness and their incurred costs. The solution approaches proposed by these two research papers are of limited applicability to manual picker-to-parts systems. Single processing times are used in both solution approaches in order to determine a sequence of batches. Therefore, they will not yield competitive results in the situation described in our paper. Won and Olafsson [28] endeavored to integrate order batching and picker routing problems together in order to minimize the travel time. They compared their proposed heuristics with other existing algorithms such as FCFS. It can be perceived that their simulation results improvement is reduced when the numbers of items for each order increase. Tsai et al. [29] attempted to solve a batch picking model considering earliness and tardiness penalty using a multiple-GA method for obtaining the best possible batch picking plans. They tried to develop a flexible model to cope with the current dynamic environment in terms of responsiveness. Upon studying their proposed method, it was determined that the orders were split which causes items in an order to be collected in different tours. Also, their proposed model allocates penalty to batches overweight (when the batch volume exceeds the picker capacity) which is not applicable in practice.

### 2.2. Picker Routing Problem

The picker routing problem is defined as the process of identifying the minimum distance which would be traveled by the order picker in a warehouse upon identifying which order should be picked first [30, 31]. There are some existing heuristics for picker routing such as return, s-shape, largest gap, and combined routing. De Koster et al. [32] tried to solve order batching problems by evaluating two groups of heuristic methods such as seed algorithms and time savings algorithms by means of s-shape and largest gap strategies as two different routing strategies. According to the obtained results, integrating the s-shape strategy and an outsized capacity pick device together with seed algorithms can yield better performance. In contrast, the largest gap strategy using a small capacity pick device gives best performance when it integrated with time savings algorithms. Ho and Tseng [33] considered the largest gap routing strategy together with a simulated annealing optimization method not only to optimize the total travel distance but also to find alternative routes that are better than ones found by just the largest gap strategy. In a research activity done by Theys et al. [34], s-shape, largest gap and some other routing heuristics were compared with the Lin-Kernighan-Helsgaun TSP heuristic. Based on their results, this TSP heuristic helps to reduce travel distance up to the rate of 48% on average. In the current research activity, a GA__TSP_ model has been used in order to solve the picker routing problem.

### 2.3. Batch Sequencing Problem

The problem of batch sequencing can be defined as finding the orders of constructed batches to be processed further. Henn and Schmid [35] used metaheuristics to solve their proposed model of order batching and sequencing to minimize the orders' tardiness. Iterated Local Search and Attribute-Based Hill Climber are the two employed metaheuristics in their model. According to different test problems, the efficiency of their proposed methods was illustrated. Provided solutions can be improved by 46% on average in comparison with the ones obtained by standard constructive heuristics such as an application of the Earliest Due Date rule. They conducted their proposed method for a maximum of 80 items. 

Other sections of this paper are as follows. First, the proposed model is described in Section 3 which covers all the aspects that are involved with it. After that, Section 4 presents the research methodology of this paper. This is followed by Section 5 which encompasses the numerical examples, results, and discussion of this research activity. Finally, Section 6 gives an end to the paper with a brief conclusion.

## 3. Model Description

The assumptions of this research are described as follows.

1. A manual picker to part system in a parallel aisle warehouse is considered in this research.
2. There is only one location for storing each type of item and vice versa.
3. Multiple picker devices are not allowed. Therefore the maximum number of pickers is only one.
4. The volume for each order should not exceed the capacity of the picker.
5. Batch size does not exceed the capacity of picker.
6. The storage size in each location is identical.
7. All customer orders are known in advance.

### 3.1. Weighted Association Rule Mining Binary Integer Clustering Model

 In this section, an association based clustering model developed by Chen and Wu [23] was modified in order to create the batch structure regarding orders' due date. First, the association between customers' demand will be calculated based on the similarity of items in orders with respect to their due date using MINWAL. Orders' due dates are considered as the weight of association rule mining. Although batching orders with higher associations generated by similarity between their items can decrease the distance travelled by order pickers, not considering the due dates would cause the orders to be batched inappropriately with different due dates. Some problems may occur by the aforementioned issue such as shortage or excess inventory costs. In order to solve this problem, the order due date has been considered as a weight of each order for calculating the support between orders. Relationship maximization of customer orders based on similar items within each order and due dates of each order can be done with the weighted association rules. Travel distance would be reduced by taking into account batching of orders with higher relationships. Other than that, higher associations can batch orders with similar due dates in order to minimize tardiness. This is followed by utilizing a binary integer programming model integrated with a clustering algorithm which assembles the orders into their respective batches.

#### 3.1.1. Weighted Association Mining for Determining Customer Order Relationships

Association rule mining can be defined as a data mining method which tries to distinguish interrelations of variables where large databases exist [36]. Some rules are involved with the association rule model where there is an association between some set of items with another set of items [37]. In order to calculate rule interestingness, support and confidence can be considered as the two most appropriate measures in association rule mining. The support for an itemset is the percentage of transactions that contain the itemset in the database. A probability shows how frequently the rule head occurs among all the groups containing the rule body which can be defined as the confidence of an association rule (body⇒head) [38, 39].

Based on the definition by Agrawal et al. [36], the association rule mining problem is addressed as follows: items are described as *I* = {*i*_1_, *i*_2_,…, *i*_*n*_} which is a set of n binary attributes. The database is represented as *D* = {*t*_1_, *t*_2_,…, *t*_*m*_} which is a set of transactions. An exclusive transaction *ID* belongs to each transaction in *D* together with a subset of the items in *I*. A rule is defined as an implication of the form *X*⇒*Y*, where *X*, *Y*⊆*I*, and *X* ⋂ *Y* = *ϕ*. The association rule, *X*⇒*Y* [support = *s*%, confidence = *c*%], holds in the item-order data *D* with confidence *c* if *c*% of Order Identifiers in *D* contain orderset *X* and also contains orderset *Y*. The rule *X*⇒*Y* has support *s* if *s*% of the item-order *D* contains both *X* and *Y*. For instance, upon batching the orders by taking into account the customer order patterns, association rule mining involves finding out the amount of support between demands of customers from the order database. In order batching, Table 1 which shows the order-item data is transposed to the item-order data form shown in Table 2 since the order associations are demanded and the product item associations is not necessary. In this example, association rule mining can perform its job to define the associations between customer orders that can be perceived from information presented in Table 2.

In the area of order batching, association rule mining was first used by Chen and Wu [23]. In their research paper, association rule mining was used to calculate the correlations between customers' demands in terms of support. They pointed out that orders with similar items should be consolidated together in order to minimize the total travel distance traversed by the picker. An Apriori algorithm has been used in their proposed method to calculate the association between orders. It should be highlighted that they only considered the relationships between orders in terms of similarities between items. However, in batch processing, each order has a due date which can be considered as the weight but this weight is not considered by any other existing method in the literature.

The classical Apriori algorithm [40] extracts binary association rules based on the downward closure property which proves that subsets of a frequent itemset are also frequent [41]. However, the Apriori algorithm cannot be applied because it cannot handle the weighted case. Therefore, in this research activity, the MINWAL algorithm is applied in order to extract the weighted supports among the pair of orders to fulfill the task of considering both the support and the weights factors. 

In order to use MINWAL [42] for mining weighted association rules, the weight should be in the range of the 0 to 1 interval. Based on the aforementioned fact, larger weights should be assigned to orders with smaller due dates in a comparison with other orders that have lots of time to be delivered in order to minimize the average tardiness of all orders. Therefore, in this study, (1) has been used to calculate the appropriate weights for each order as following:

(1) $$\begin{array}{c} \text{Weight}\left(O_{i}\right)=e^{\left(-0.1*\beta *d_{O_{i}}/\text{Maximume}\;\text{Due}\;\text{Date}\right)}, \end{array}$$

where *O*_*i*_ denotes an order and *d*_*O*_*i*__ refers to the due date of order *O*_*i*_. *β* is a constant value that is used to normalize the due dates which is considered as 30 (30 minute) in this study. Also, the maximum due date of the orders was used in order to provide an appropriate interval for the weights. For example, the weight of each order is tabulated in Table 3 which is calculated by (1).

Given a set of orders *O* = {*o*_1_, *o*_2_,…, *o*_*m*_}, a weight *w*_*j*_ for each order *o*_*j*_, within the values of 0 to 1 is assigned by (1) via its due date, where *j* = {1, 2,…, *m*} shows the importance of it. The weighted support for the weighted association orders of *O*_1_ and *O*_2_ as *O*_1_⇒*O*_2_ is defined, if the weighted support of such a pair of orders is no less than the minimum weighted support (minsup) threshold. In this study, the minimum support threshold is considered as 0 in order to identify all the associations among orders. Equation (2) shows the support of an association for any pair of orders as following:

(2) $$\begin{array}{c} \left(\sum \underset{w_{j}\in \left(O_{1}\cup \;O_{2}\right)}{\sum }w_{j}\right)*\text{Support}\left(O_{1}\cup O_{2}\right). \end{array}$$

Algorithm 1 shows the MINWAL steps in order to mine weighted association rules. This algorithm consists of 6 subroutines such as, Search, Counting, Join, Prune, Checking and Rules. The search subroutine provides the size of the largest itemsets in database. Counting is responsible for counting every 1-itemset inside the database. The Join step is responsible for generating the *k*-itemsets (*Ck*) from (*k* − 1)-itemsets (*Ck* − 1). The Prune step removes inappropriate itemsets which do not exist. The Checking step updates the count of *k*-itemsets inside the transaction and prunes itemsets which do not meet the minimum support threshold. In the Rules step, every association rule will be extracted.  Table 4 shows the notations of Algorithm 1.

#### 3.1.2. Association Based Binary Integer Programming for Orders Batching

In order to create the batch structure, a binary integer clustering model which has been developed by Chen and Wu [23] was applied. They developed a binary integer programming model for maximizing similarity between orders in terms of support in order to minimize the total traveling distance. In this research activity, support between orders was calculated based on weighted association mining using MINWAL which was described in Section 3.1.1. The binary integer model for order batching is described as follows.

*Parameters*

*S*_*ij*_ Support between order *i* and *j* determined by MINWAL
*C*_*v*_ Capacity of picking vehicle
*V*_*i*_ Weight of order *i*
K Number of batches
*X*_*ij*_ {1 if  order  i  is  assigned  to  batch  j;0 otherwise;         
*Y*_*i*_ {1 if  order  j  is  chosen  as  a  batch  median; 0 otherwise;             
  (3) Maximize    ∑i=1N∑j=1NSij∗Xij                
  (4)   Subject  to    ∑j=1NXij=1 for  i,j=1,2…,N
  (5) $$\begin{array}{c} X_{ij}\leq Y_{j}\;\;\text{for}\;i,j=1,2\ldots ,N \end{array}$$
  (6) $$\begin{array}{c} \overset{N}{\underset{j=1}{\sum }}Y_{j}=K \end{array}$$
  (7) $$\begin{array}{c} \overset{N}{\underset{i=1}{\sum }}V_{i}X_{ij}\leq C_{v}\;\text{for}\;j=1,2\ldots ,N \end{array}$$
  (8) $$\begin{array}{c} X_{ij}=0,1\;\text{for}\;i,j=1,2\ldots ,N \end{array}$$
  (9) $$\begin{array}{c} Y_{j}=0,1\;\text{for}\;j=1,2\ldots ,N. \end{array}$$

In the current model, the objective function (3) maximizes the sum of support from all orders to their relevant batch medians as *K* orders are selected as batch medians. This model will maximize the support between orders in the batches. Membership of each order in just one batch would be guaranteed by constraint (4). The number of batches with *K* is limited using constraints (5) and (6). The total quantity in a tour is also represented as a limitation in constraint (7). It cannot exceed the capacity of order picker *C*_*v*_. For the order batching model to have a binary solution would be ensured by constraints (8) and (9). For calculating the initial numbers of batches (10) will be used where ∑*V*_*i*_ represents total volume of all orders as following:

(10) $$\begin{array}{c} K_{\text{initial}}=\;\left\lceil \frac{\sum V_{i}}{C_{v}\;}\right\rceil . \end{array}$$

### 3.2. Picker Routing

#### 3.2.1. Genetic Algorithms

In this research, GAs which were introduced by Holland [43] have been used in order to solve the picker routing and batch sequencing problem. The biological evolution process is used as a base fact in a GA for problem solving to a great extent. The main steps of a GA can be described as follows [44, 45]:

- populating a series of solutions,
- solutions assessment,
- picking the most appropriate solutions,
- generating new solutions based on some genetic operation.

In step (iii), selection forms the main part of a GA. The bad solutions are removed by selection and the good ones remain. Random generation over the feasible or infeasible solution space of a problem is used as a basis for creating the population. Each solution is evaluated using the fitness function. Selection would be done according to certain selections of fitness together with the probability that is assigned to it. Generating a fresh population and a better solution are involved with recombination of the individual solution. Crossover and mutation are the most used GA operators for generating new offspring from the old generation of parents. Parameters of the GA are originated from the probabilities of mutation and crossover. Exploration of the sample space is diversified by these operators. In this research, the GA has been utilized in two steps. First, the GA has been used in order to solve the picker routing problem. Consider that in the warehouse a set of items should be picked by a picker in one tour. Therefore, the picker routing problem in the warehouse could be categorized as a TSP. The goal in the picker routing problem as a TSP in the warehouse is to find an optimal path for minimizing the travel distance traversed by the picking machine where each node is visited at least once. In this research, for a given number of itemset in a batch, GA__TSP_ has been used to find the minimum travel distance which is traversed by the picking machine. Second, a GA has been utilized in order to sequence all batches, namely, GA__sequencing_ which would be further processed. The batches are sequenced based on a fitness function which minimizes the tardiness of all orders. The detailed procedures of GA__TSP_ and GA__sequencing_ are illustrated in Sections 3.2.3 and 3.2.4.

#### 3.2.2. Warehouse Layout

The warehouse discussed throughout this research is assumed to have a 3D multi-parallel-aisle warehouse layout. The picker will start the tour from the depot which is located in front of the leftmost aisle and returns back to it. A picker leaves the depot. After that, it travels to a certain buffer spot where it picks the requested order(s) and puts the order(s) into a certain picker. Then, it continue on its way to another spot. These procedures would be repeated again and again so that all the requested orders are picked. Then, it is time for the picker to return to the depot.

**Figure 1:**
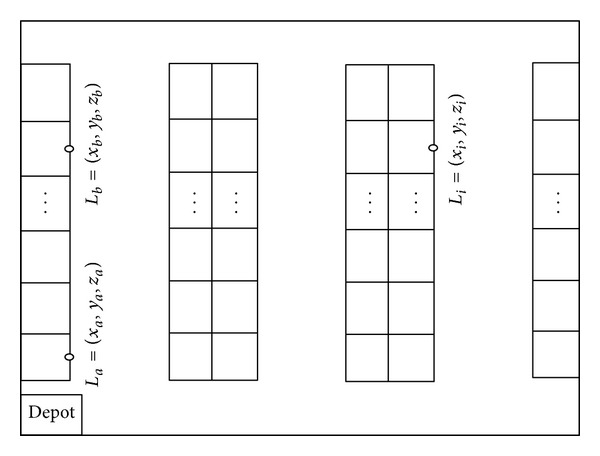
Warehouse layout.

The graphic layout of the warehouse zone involved in this research activity is pictorially displayed in Figure 1. It shows the *x* and *y* dimensions of the 3D warehouse layout. Each item in the warehouse has a specific location. For example, the location of item *i* can be addressed by *L*_*i*_ = (*x*_*i*_, *y*_*i*_, *z*_*i*_). Consider two locations as *L*_*a*_ = (*x*_*a*_, *y*_*a*_, *z*_*a*_) and *L*_*b*_ = (*x*_*b*_, *y*_*b*_, *z*_*b*_). If two locations are located in the same sub-aisles of the same block, the distance between these two items locations, (*D*_*a*,*b*_), will be calculated using

(11) $$\begin{array}{c} D_{a,b}=\left|x_{a}-x_{b}\right|+\left|y_{a}-y_{b}\right|+\left|z_{a}-z_{b}\right|. \end{array}$$

Equation (11) is not applicable if the two storage locations belong to different subaisles that are from the same block [34]. In order to solve this problem, distances of all possible paths between two locations have been calculated and the shortest one was considered. 

#### 3.2.3. GA_**_TSP**_ for the Picker Routing Problem

 In order to determine the optimal route of a picker related to a given set of items in a batch, a GA has been used to solve the TSP problem to minimize travelling time. In a GA, each solution is considered as a chromosome. The steps of GA__TSP_ are described as follows:

1. for each batch establishing the structure of a chromosomes,
2. generate initial feasible chromosomes,
3. evaluate each chromosome based on the GA__TSP_ fitness function,
4. apply the Selection operation,
5. apply the Crossover operation,
6. apply the Mutation operation,
7. evaluate each offspring based on the fitness function,
8. go to step 4, if the termination criterion is not satisfied, otherwise terminate and return the best solution as the optimal path.

**Figure 2:**
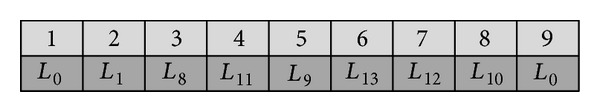
A feasible chromosome encoding in GA__TSP_.

At the first step of GA__TSP_, the structure of the chromosome should be established. In the GA__TSP_, the location of an item that should be visited is presented by the value of a gene. Accordingly, the sequence of visiting the item location is denoted by the order of a gene in a chromosome. Therefore, the total number of items locations in a specific batch is considered equal to the length of a chromosome. For example, if the length of a chromosome is nine it means nine places should be visited by the picker as shown in Figure 2. If *L*_*i*_ represents the item location in the warehouse, *i* = {1,…, *n*}, it shows that the picker leaves the depot (*L*_0_ denotes the location of a depot), goes to *L*_1_, *L*_8_, *L*_11_, *L*_9_, *L*_13_, *L*_12_, *L*_10_, and then comes back to the depot.

In the second step, the initial number of feasible chromosomes should be generated randomly for each batch. The number of initial chromosomes could be different based on the complexity of the problem. It should be considered that the first and the last gene of each chromosome should be assigned individually to the location of a depot. In step three, each chromosome will be evaluated based on the GA__TSP_ fitness function which is formulated as follows:

(12) $$\begin{array}{c} \text{fitness}=\mathrm{Min}\;\overset{n-1}{\underset{j=1}{\sum }}D_{j,j+1}, \end{array}$$

where *D*_*j*,*j*+1_ denotes the distance between two subsequent genes in a chromosome, *j* = 1,…, *n* − 1 represents the order of the genes, and *n* is number of genes.

Subsequently, step four is involved with the selection operation. In each iteration, the selection operator uses fitness values to select the parents of the next generation. In this research, the roulette wheel selection policy has been used to guarantee that the most appropriate pairs of chromosomes have been selected to generate offspring. It means that a higher probability of being selected to create a new population will be assigned to the chromosomes with higher fitness values.

**Figure 3:**
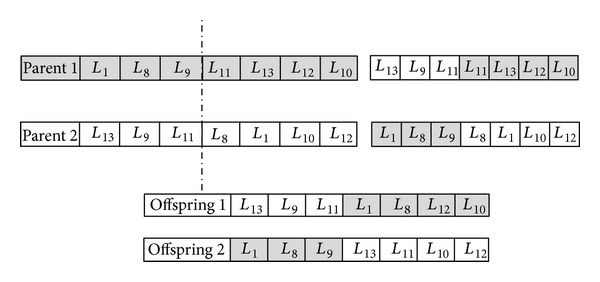
Crossover operation.

Steps five and six are involved with conducting the crossover and mutation operation. The construction of the offspring is realized by the crossover and the diversity of the individuals is maintained by the mutation operator. Blocks of genes between chromosomes are traded by means of the crossover operation in which the exploitation of a particularly profitable portion of the parameter space is allowed to the block. Here, permutation order based crossover (POP) as a variation of the distinguished order crossover has been used. As shown in Figure 3, the original idea is about choosing two parents and a point for cutting. The first portion of offspring 2 contains the first portion of parent 1 up to the cut point. To form the second portion of offspring 2, consider all genes in parent 2 except those genes that already exist in the first portion of the offspring 2.

**Figure 4:**
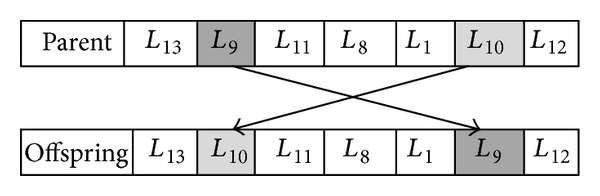
Mutation operation.

As shown in Figure 4, two positions are randomly selected in a chromosome and exchanged with each other; namely, the order based mutation approach, SWAP, is adopted for solving GA__TSP_. Step six is followed by step seven which includes the evaluation of each offspring that has been generated in the previous step based on the GA__TSP_ fitness function. In step eight, termination criteria will be checked. If the termination condition is met, then the process will be terminated. After that, the best chromosome will be selected as the optimal solution. Otherwise, the selection process is to be conducted again to generate a new population.

#### 3.2.4. GA for Sequencing the Batches

**Figure 5:**
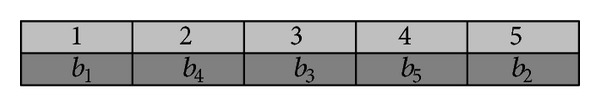
Chromosome structure.

Sequencing the batches should be conducted in order to minimize the average tardiness of all orders. The problem of batch sequencing can be defined as finding the orders of batches which should be processed further. For this purpose, the GA__sequencing_ method has been utilized. In this research, all of the batches which were constructed using binary integer programming and GA__TSP_ will be sequenced by using the GA. The population generation, selection, mutation, and stopping threshold in the GA__sequencing_ are identical to those in the GA__TSP_. However, the fitness function and chromosome structure are different than that of GA__TSP_. In contrast with GA__TSP_ chromosomes structure, batch number is presented by the value of a gene. Accordingly, the sequence of batch is denoted by the order of a gene in a chromosome. For instance, Figure 5 shows the chromosome structure. If *b*_*i*_ ∈ *B* denotes a set of batches, Figure 5 shows that *b*_1_, *b*_4_, *b*_3_, *b*_5_, *b*_2_ should be processed, respectively. Therefore, the length of a chromosome is equal to the number of the batches. The GA__sequencing_ fitness function for chromosome *y* is described in

(13) $$\begin{array}{c} {\text{fitness}}_{y}=\frac{1}{N}\;\overset{n}{\underset{k=1}{\sum }}\;\underset{i\in b_{k}}{\sum }CT\left(b_{k}\right)-Du\left(o_{i}\right), \end{array}$$

(14) $$\begin{array}{c} CT\left(b_{k}\right)=CT\left(b_{k-1}\right)+{\text{P}}_{\text{t}}\left(b_{k}\right)\text{,} \end{array}$$

(15) $$\begin{array}{c} {\text{P}}_{\text{t}}\left(b_{k}\right)=\frac{\text{Opt}\left(b_{k}\right)}{VS}, \end{array}$$

where *N* denotes for the total number of orders. *k* = {1,…, *n*} represents the batch sequence in which *n* is the total number of batches. *o*_*i*_ stands for the order *i* in which *i* ∈ *b*_*k*_. *b*_*k*_ denotes the batch in *k*th position of a sequence for specific chromosome. *CT*(*b*_*k*_) stands for the completion time of a batch in the *k*th position of a sequence. *Du*(*o*_*i*_) is the symbol for due date of order *i* which belongs to *b*_*k*_. P_t_(*b*_*k*_) is the process time of *b*_*k*_. Opt(*b*_*k*_) is the optimal path solution for *b*_*k*_. Finally, *VS* is the moving speed of the picker.

## 4. Research Methodology

**Figure 6:**
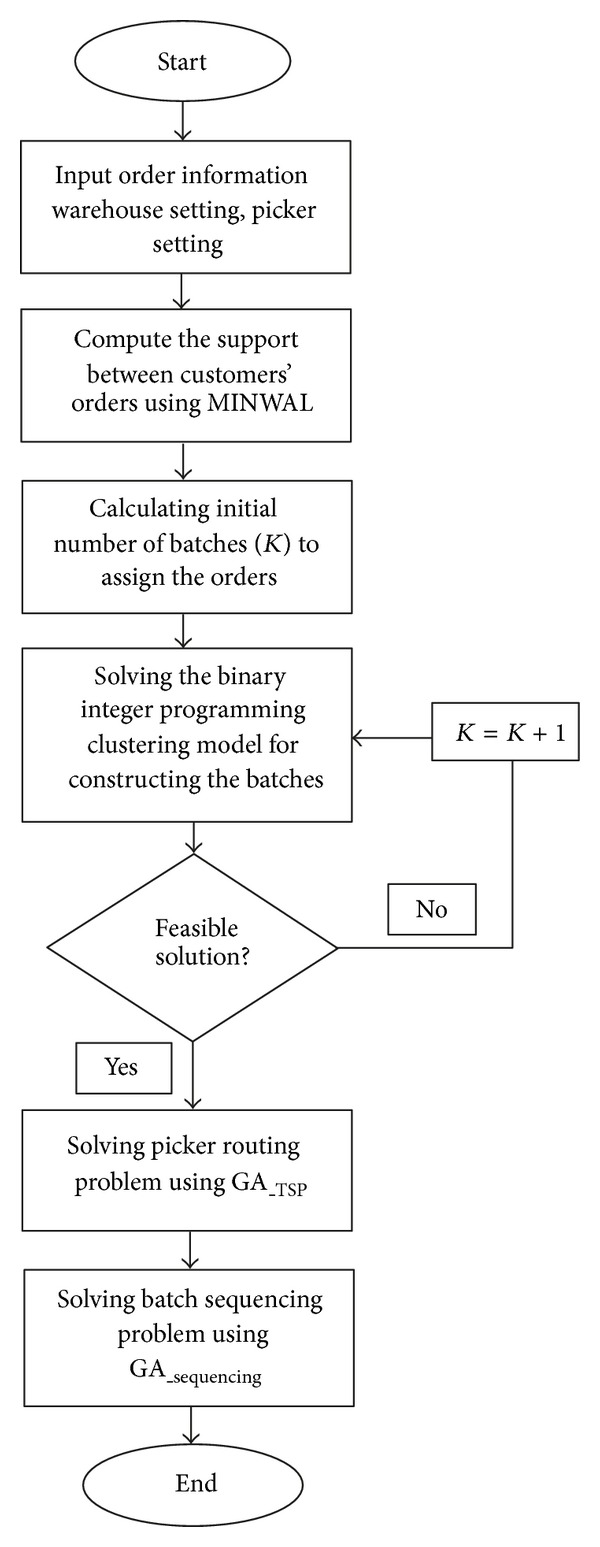
Methodology framework.

In this research, the proposed methodology consists of five steps. These steps are described as follows and shown pictorially in Figure 6.

**Step 1**

It is involved with utilizing the MINWAL algorithm in order to determine the associations between orders in terms of support considering their due dates. In this step, an order due date will be considered as the weight of an order. It means orders that are similar in items and due date should have more support for being batched together.

**Step 2**

It encompasses determining the initial number of batches. In this step, the initial number of batches will be calculated by (10).

**Step 3**

It includes the problem of modeling and solving; first, the binary integer programming model for order clustering and batching will be modeled. Next, this model will be solved in order to determine the structures of batches.

**Step 4**

It is about the picker routing problem. The optimal path for a picker within each batch will be determined by solving a TSP using GA.

**Step 5**

It is about sequencing the batches based on the fitness function which minimizes the average tardiness of all orders. In this step, GA algorithm will be utilized in order to solve the batch sequencing problem regarding the fitness function.

## 5. Computational Experiments and Results

### 5.1. Test Problems and Parameter Setting

 In this section, five numerical test problems with different settings and parameters have been carried out in order to illustrate the proficiency of the proposed method with respect to average tardiness of all orders. Table 5 summarizes the description of these test problems. This table includes information with regard to number of items, number of orders, capacity of the picker, total weight of all the items in an order, and minimum number of batches. 

In these test problems, the quantity of items, quantity of orders, capacity of the picker device (*C*_*v*_), and amount of each item are predetermined. Each order contains a certain quantity of items and also each item has a certain quantity. These quantities are produced randomly. The distribution function type for the number of items in each order is Normal [5, 10]. Also, the distribution function type for each item quantity in an order is uniform [1, 10]. The picker moving speed is set as *VS* = 2 (m/s). The warehouse has 3000 item location. The GA control parameters for solving the TSP and sequencing problem are tabulated in Table 6.

All of the proposed approaches are coded using MATLAB version 7.9.0 and Java 1.5 programming language. Also, the IBM ILOG CPLEX optimization studio version 12.4 software was utilized jointly with MATLAB in order to facilitate the process of solving the binary integer programming problem. Examples are run by means of a computer featured by 4 Gigabytes random access memory (RAM) and a 2.20 Gigahertz Intel processor (T6600).

### 5.2. Computational Results and Discussion

In order to reveal the competency of the proposed solution approach, it was compared with the other existing methods in the literature. One of the latest published research activities in the literature which deals with minimization of tardiness has been conducted by Henn and schmid [35]. They utilized the Earliest Due Date (EDD) approach as their constructive algorithm for comparing with their proposed approaches. They proposed two heuristics. Iterated Local Search and Attribute-Based Hill Climber are the two employed metaheuristics in their model. According to different test problems, the efficiency of their proposed methods was illustrated. Their obtained results from their proposed approaches showed a 46% improvement on tardiness compared to EDD. They used an s-shape and largest gap routing strategy for their proposed method. In order to make a reasonable comparison with their proposed method, EDD was integrated with the s-shape routing strategy and GA__sequencing_. Then, based on improvement on EDD, we can compare our proposed method with their proposed method. In the EDD approach, due dates play an important role in sorting all orders. Based on an ascending sequence, assigning the orders to batches ensures that no capacity violation can happen for the picking device.

For the warehouse condition which is described in Section 3.2.3, our proposed approach, EDD integrated with GA__TSP_ and GA__sequencing_ and EDD integrated with s-shape algorithm and GA__sequencing_ have been evaluated in terms of average tardiness for all orders (*T*) which is calculated based on (13), number of batches (*K*), total process time (P_t_), and average process time for each batch (*A*_P__t_). All of the GA setting for the TSP and sequencing problems are considered the same for all methods. Also, the settings which have been mentioned in Section 5.1 were considered for all of the compared methods.

The results for all of the test problems for the three approaches are tabulated in Tables 7, 8, and 9. As it is illustrated in Table 10, EDD integrated with GA__TSP_ and GA__sequencing_ has a 31.76% improvement on average in terms of average tardiness of all orders than EDD integrated with the s-shape algorithm and GA__sequencing_. It can be perceived that GA__TSP_ performs better than s-shape. Also, the ATGH method has a 68.11% improvement in average tardiness off all orders than EDD integrated with s-shape algorithm and GA__sequencing_. Consequently, the ATGH method achieves much improvement against Henn and Schmid's [35] proposed method. According to Tables 7 and 8, it is also worth to mention that GA__TSP_ performs better than the s-shape routing strategy in terms of process time. The improvements which have been achieved in this research could be justified based on the following reasons.

The problem of order batching deals with several significant drivers in which the number of batches can be considered as a key factor [23]. As a justification to this matter, it is worth to mention that total travel distance may not decrease directly due to a decline in the number of batches. But a decline in the number of batches will increase batch sizes which may lead to an increase in the distance travelled by order picker. However, an advanced exploitation of picker capacity to load efficiently may reduce the number of constructed batches together reducing the total travel distance. Improving the total travel distance traversed by the picker can minimize the average tardiness of all orders. In terms of number of the batches according to Tables 7, 8, and 9, it can be perceived that our proposed method assigns the orders to the batches optimally. The ATGH method assigns the orders to batches based on their item similarity and their due dates and it uses the picker capacity efficiently. But the EDD method allocates the orders to batches based on an ascending sequence of their due date and it should be ensured that no capacity violation will happen for the picking device which may lead to an inefficient order batching. To highlight the problem, an example can be made based on the results tabulated in Tables 7 and 8. The number of batches for example 1 in our method is 11 in comparison with 13 obtained from EDD method. 13 batches can be justified based on this scenario that two subsequent orders, say, the first order is 100 kg and the other is 150 kg, cannot be batched together due to violated batch capacity which is set to be 200 kg. The number of batches would increase due to the aforementioned issue which affects the total process time to have an increasing manner. This situation will rarely happen in the ATGH method because of the weighted association based binary integer programming utilized which batches the orders in order to maximize the association between them which is calculated based on the item similarity of orders and the weight of their corresponding due dates. The ATGH method uses the picker capacity efficiently.

Obviously, one of the factors that can influence the completion time and the average tardiness of all orders is the processing time of the orders. Reducing the processing time and travel time for constructing a batch is an outstanding fact in reducing the costs and minimizing delays of the customer responses. In our proposed model, orders with similar items are consolidated together. Therefore, batching orders with more similarity can reduce the distance traversed by the picker facility. Also, the due date of each order is defined as a weight parameter in calculating the association between orders in terms of support which was done for considering the due dates in our model. Thereupon, the model considers due date and the process time in an integrated manner which simultaneously affected the average tardiness of all orders. This spoken issue is different in the EDD approach in which the orders' due dates are just considered. Example 5 can be considered as evidence of this matter in which the total and average process time for ATGH is lower than EDD integrated with GA__TSP_ and GA__sequencing_ with respect to the fact that number of constructed batches for both approaches are happened to be equal after obtaining the results.

## 6. Conclusion

One of the most important concerns of warehouse managers is involved with finding the best optimal and cost efficient way to pick orders placed by customers in order to be known as a responsible company with satisfied customers in terms of on time delivery. In the current research activity, order batching, picker routing, and batch sequencing problems of warehouse processes were addressed which are to be solved jointly as they are mixed up with many manufacturing industries. Mathematical solutions of these NP-hard problems might be available for problems that are small in nature. But, these kinds of solutions may not be applicable to be considered by warehouse managers. Hence, we presented novel solution approach, namely, ATGH, to solve this issue in accordance with minimizing the average tardiness of all customers' orders which can be considered as a major objective of this research activity. A weighted association rule mining algorithm, namely, MINWAL, was utilized to determine the customers' orders association regarding similarity between items and orders' due dates. Actually, in our proposed method the due date of each order is defined as a weight parameter in calculating the association between orders. So, orders with similar items and due dates have more chance to be in a same batch. This issue is not considered in most of the research activities in the literature related to our work and considering this issue in the order batching problem is the main contribution of our research activity. The previous step was followed by a binary integer clustering model in order to solve the order batching problem which maximizes the similarity between orders in terms of support. After that, a GA was applied to solve a TSP in order to find the optimal routing path.

Finally, a batch sequencing problem was addressed in conjunction with a GA. 

The other research objective of this research addresses whether the proposed ATGH algorithm can further improve the solution quality for a multiple parallel aisle warehouse with manual picking system. Hence, comparisons were made considering ATGH, EDD integrated GA__TSP_ and GA__sequencing_, and EDD integrated s-shape and GA__sequencing_ algorithms. As demonstrated by the obtained results, ATGH comes out to be the most striking solution approach in terms of tardiness. In spite of the fact that considering all customer orders, as discussed in this paper, would lead to have a static case, being involved with dynamic cases in which different points of time are assigned to customer's orders arrival would be beneficial. So, there might be some potential room of research to extend our discussed approach with respect to dynamic orders.

## Figures and Tables

**Algorithm 1:**
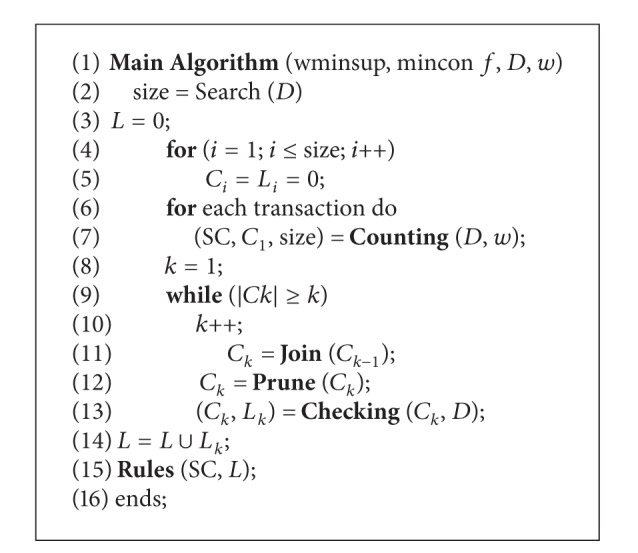
MINWAL algorithm [42].

**Table 1:** Order-item data.

Order	Items	Due date
*O*_1_	A(3) B(4)	10
*O*_2_	A(2) C(3)	30
*O*_3_	A(1) B(3)	5
*O*_4_	A(2) B(4) C(2)	50

**Table 2:** Item-order data.

Item	Order
A	*O*_1_, *O*_2_, *O*_3_, *O*_4_
B	*O*_1_, *O*_3_, *O*_4_
C	*O*_2_, *O*_4_

**Table 3:** Weight of each order.

Order	Weight
*O*_1_	0.5827
*O*_2_	0.1979
*O*_3_	0.7634
*O*_4_	0.0672

**Table 4:** Notations of Algorithm 1.

*D*	The database
*w*	The set of item weights
*L*_*k*_	Set of large *k*-itemsets
*C*_*k*_	Set of *k-*itemsets which may be *k*-subsets of large *j*-itemsets for *j* ≥ *k*
SC (*X*)	No. of transactions containing itemset *X*
wminsup	Weighted support threshold
minconf	Confidence threshold
Size	Maximum possible large weighted itemsets

**Table 5:** Test problems description.

	Example 1	Example 2	Example 3	Example 4	Example 5
Number of orders	40	60	80	100	150
Number of items	80	120	160	200	200
Capacity of picker	200	200	300	400	550
Total weight	2212	3668	4393	6110	8369
Minimum number of batch	11	19	15	16	16
Due dates range	60	90	120	180	240

**Table 6:** GA control parameters.

	GA__TSP_	GA__sequencing_
Population size	500	200
Cross over strategy	Permutation order based crossover (POP)	Permutation order based crossover (POP)
Cross over probability	0.8	0.8
Mutation strategy	SWAP	SWAP
Mutation probability	0.2	0.2
Iteration number	10000	10000
Parent selection method	Roulette wheel	Roulette wheel

**Table 7:** Computational results of ATGH.

	Example 1	Example 2	Example 3	Example 4	Example 5
*T*	5.6204	29.948	9.0202	1.63	5.8898
P_t_	60.5833	121.7833	105.833	121.033	133.9
*K*	11	19	15	16	16
*A*_P_t__	5.5076	6.409	7.055	7.564	8.368

**Table 8:** Computational results of EDD integrated with GA__TSP_ and GA__sequencing_.

	Example 1	Example 2	Example 3	Example 4	Example 5
*T*	12.4550	42.976	18.222	5.981	15.47
P_t_	66.7333	133.516	116.083	131.85	141.916
*K*	13	21	16	17	16
*A*_P_t__	5.1333	6.357	7.255	7.775	8.869

**Table 9:** Computational results of EDD integrated with s-shape algorithm and GA__sequencing_.

	Example 1	Example 2	Example 3	Example 4	Example 5
*T*	16.51	62.677	28.25	13.365	17.593
P_t_	75.266	166.36	133.23	143.33	149.26
*K*	13	21	16	17	16
*A*_P_t__	5.789	8.92	8.33	8.431	9.32

**Table 10:** Comparison results.

	EDD + s-shape + GA__sequencing_	EDD + GA__TSP_ + GA__sequencing_		ATGH	
	Tardiness (min)	Tardiness (min)	Improvement (%)	Tardiness (min)	Improvement (%)
(1)	16.51	12.4550	24.56%	5.6204	65.96%
(2)	62.677	42.976	31.43%	29.948	52.22%
(3)	28.25	18.222	35.50%	9.0202	68.07%
(4)	13.365	5.981	55.25%	1.627	87.83%
(5)	17.593	15.47	12.06%	5.8898	66.52%

Average improvement			31.76%		68.11%
